# Supplementary material for: Sampling Variation of RAD-Seq Data from Diploid and Tetraploid Potato (Solanum tuberosum L.)
Source: Plants (Basel). 2021 Feb 7;10(2):319. doi: 10.3390/plants10020319 (PMC7915145; doi:10.3390/plants10020319)
Supplement: Supplementary file 1 [file plants-10-00319-s001.pdf]

Supplementary Table S1 A complete list of all Illumina adapters used in the optimized RAD-seq study.

a) Sample-specific adapters for EcoRI cut site ligation

Adapters	Nucleotide sequences
EcoRI_P1.1	ACACTCTTTCCCTACACGACGCTCTTCCGATCTACTGG
EcoRI_P1.2	ACACTCTTTCCCTACACGACGCTCTTCCGATCTAGCTA
EcoRI_P1.3	ACACTCTTTCCCTACACGACGCTCTTCCGATCTATACG
EcoRI_P1.4	ACACTCTTTCCCTACACGACGCTCTTCCGATCTCGATC
EcoRI_P1.5	ACACTCTTTCCCTACACGACGCTCTTCCGATCTCGTAC
EcoRI_P1.6	ACACTCTTTCCCTACACGACGCTCTTCCGATCTCTGAT
EcoRI_P1.7	ACACTCTTTCCCTACACGACGCTCTTCCGATCTGAGTC
EcoRI_P1.8	ACACTCTTTCCCTACACGACGCTCTTCCGATCTGCTGA
EcoRI_P1.9	ACACTCTTTCCCTACACGACGCTCTTCCGATCTGTCGA
EcoRI_P1.10	ACACTCTTTCCCTACACGACGCTCTTCCGATCTTACCG
EcoRI_P1.11	ACACTCTTTCCCTACACGACGCTCTTCCGATCTGCATG
EcoRI_P1.12	ACACTCTTTCCCTACACGACGCTCTTCCGATCTTCAGT
EcoRI_P2.1	[Phos]AATTCCAGTAGATCGGAAGAGCGTCGTGTAGGGAAAGAGTGT
EcoRI_P2.2	[Phos]AATTTAGCTAGATCGGAAGAGCGTCGTGTAGGGAAAGAGTGT
EcoRI_P2.3	[Phos]AATTCGTATAGATCGGAAGAGCGTCGTGTAGGGAAAGAGTGT
EcoRI_P2.4	[Phos]AATTGATCGAGATCGGAAGAGCGTCGTGTAGGGAAAGAGTGT
EcoRI_P2.5	[Phos]AATTGTACGAGATCGGAAGAGCGTCGTGTAGGGAAAGAGTGT
EcoRI_P2.6	[Phos]AATTATCAGAGATCGGAAGAGCGTCGTGTAGGGAAAGAGTGT
EcoRI_P2.7	[Phos]AATTGACTCAGATCGGAAGAGCGTCGTGTAGGGAAAGAGTGT
EcoRI_P2.8	[Phos]AATTTCAGCAGATCGGAAGAGCGTCGTGTAGGGAAAGAGTGT
EcoRI_P2.9	[Phos]AATTTCGACAGATCGGAAGAGCGTCGTGTAGGGAAAGAGTGT
EcoRI_P2.10	[Phos]AATTCGGTAAGATCGGAAGAGCGTCGTGTAGGGAAAGAGTGT
EcoRI_P2.11	[Phos]AATTCATGCAGATCGGAAGAGCGTCGTGTAGGGAAAGAGTGT
EcoRI_P2.12	[Phos]AATTACTGAAGATCGGAAGAGCGTCGTGTAGGGAAAGAGTGT

The unique 5bp barcodes are highlighted in yellow.

b) Universal adapters for MspI cut site ligation

Adapters	Nucleotide sequence
MspI_P1	[Biotin]GTGACTGGAGTTCAGACGTGTGCTCTTCCGATCT
MspI_P2	[Phos]CGAGATCGGAAGAGCGAGAACAA
